# Niosomal formulation of mefenamic acid for enhanced cancer targeting; preparation, characterization and biodistribution study using radiolabeling technique

**DOI:** 10.1007/s00432-023-05482-8

**Published:** 2023-11-20

**Authors:** Mona A. Shewaiter, Adli A. Selim, Hassan M. Rashed, Yasser M. Moustafa, Shadeed Gad

**Affiliations:** 1 Department of Pharmaceutics and Industrial Pharmacy, Faculty of Pharmacy, Sinai University, Kantara, Egypt; 2 Labeled Compounds Department, Hot Laboratories Center, Egyptian Atomic Energy Authority, Cairo, Egypt; 3 Department of Pharmacology and Toxicology, Faculty of Pharmacy, Suez Canal University, Ismailia, Egypt; 4 Department of Pharmaceutics and Industrial Pharmacy, Faculty of Pharmacy, Suez Canal University, Ismailia, Egypt

**Keywords:** Mefenamic acid, Niosomes, Anticancer, ^131^I, Radiokinetics

## Abstract

**Background:**

This work aimed to prepare niosomal formulations of an anticancer agent [mefenamic acid (MEF)] to enhance its cancer targeting. ^131^I was utilized as a radiolabeling isotope to study the radio-kinetics of MEF niosomes.

**Methods:**

niosomal formulations were prepared by the ether injection method and assessed for entrapment efficiency (EE%), zeta potential (ZP), polydispersity index (PDI) and particle size (PS). MEF was labeled with ^131^I by direct electrophilic substitution reaction through optimization of radiolabeling-related parameters. In the radio-kinetic study, the optimal ^131^I-MEF niosomal formula was administered intravenously (I.V.) to solid tumor-bearing mice and compared to I.V. ^131^I-MEF solution as a control.

**Results:**

the average PS and ZP values of the optimal formulation were 247.23 ± 2.32 nm and − 28.3 ± 1.21, respectively. The highest ^131^I-MEF labeling yield was 98.7 ± 0.8%. The biodistribution study revealed that the highest tumor uptake of ^131^I-MEF niosomal formula and ^131^I-MEF solution at 60 min post-injection were 2.73 and 1.94% ID/g, respectively.

**Conclusion:**

MEF-loaded niosomes could be a hopeful candidate in cancer treatment due to their potent tumor uptake. Such high targeting was attributed to passive targeting of the nanosized niosomes and confirmed by radiokinetic evaluation.

## Introduction

Cancer is a condition in which cells proliferate uncontrollably and produce aggressive malignancies, which results in millions of deaths annually (Anand et al. 2022). Chemotherapy is a widely used type of cancer treatment that depends on the use of toxic substances and medications to kill cancerous cells (Anand et al. 2022; Zugazagoitia et al. 2016). High dose of chemotherapy results in a variety of side effects and damage to other healthy cells. Additionally, it has been well documented that cancer cells develop drug resistance after prolonged drug exposure (Abbas and Rehman 2018; Kumar et al. 2022). As a result, avoiding healthy cells and specifically targeting malignant ones has been the main aim of cancer research. For this purpose, the new drugs are being developed into novel nanosystems (Yao et al. 2020; Edis et al. 2021).

Chronic inflammation raises the risk of various cancer types, indicating that inflammation and cancer are inextricably linked. Consequently, reducing inflammation may be an effective strategy for both cancer treatment and prevention. Numerous non-steroidal anti-inflammatory drugs (NSAIDs) are used as anticancer medications (Zappavigna et al. 2020; Kay et al. 2019). Additionally, they have been discovered to improve and accelerate tumor radio-response (Bernardi et al. 2008; Wong 2019).

MEF is an NSAID that is commonly used to treat headache, tooth pain, and osteoarthritis. It is classified as a class II biopharmaceutical that has low water solubility and high permeability (Wichianphong and Charoenchaitrakool 2018). The ability of MEF to inhibit the cyclooxygenase-2 (COX-2) enzyme, as an important component of the inflammatory process, is part of its mechanism of action (Hosseinimehr et al. 2019). MEF inhibits the multiplication of cancer cells such as those seen in in-vitro anti-proliferative assays for liver and colon cell lines (Altay et al. 2019). MEF, along with other fenamates, has demonstrated an anticancer effect also in cervical-uterine (Soriano-Hernandez et al. 2015) and prostate cancer cells (Soriano-Hernández et al. 2012). But it has also been demonstrated in tumors from animal models of colon cancer (Seyyedi et al. 2022), and its anti-cancer effect has even been tested in human clinical trials in prostate cancer(Guzman-Esquivel et al. 2020).

The activation of the caspases-3 pathway, inhibition of hyaluronic acid synthesis and cyclooxygenase inhibition are among the hypothesized mechanisms underlying MEF’s anticancer action (Woo et al. 2004). Suppression of COX-2, which is usually overexpressed in cancer tissues, by NSAIDs has been suggested as a cancer treatment. Pro-inflammatory cytokines are essential in cancer cell proliferation. So, the inflammatory environment is associated with DNA damage and death in normal cells (Hosseinimehr et al. 2015; Zha et al. 2001). It is indicated that NSAIDs decrease cancer growth by blocking such inflammation process (Wong 2019). In addition, MEF has been shown to selectively inhibit the NLRP3 inflammasome and the release of IL-1β, independent of its COX-mediated anti-inflammatory activity (Guzman-Esquivel et al. 2022), which is also a mechanism that could have an antineoplastic effect (Guzman-Esquivel et al. 2022; Blevins et al. 2022). MEF not only has anticancer activity but also protects normal cells from ionizing radiation-induced DNA damage in vitro (Hosseinimehr et al. 2015).

Nanotechnology was applied for the formulation of chemotherapeutic drugs to increase their therapeutic efficacy (Faheela and Malathi 2022; He et al. 2019). Nanocarriers can accumulate specifically in cancerous tissue due to the enhanced permeability and retention effect (EPR). This passive process of drug accumulation at tumor sites necessitates extended drug circulation for effective drug delivery (Edis et al. 2021; Xiao et al. 2021; Navya et al. 2019). Numerous carriers, including immunoglobulin, serum proteins, synthetic polymers, liposomes, microspheres, erythrocytes and niosomes have been used for enhancing drug targeting (Hua et al. 2015; Parveen et al. 2012; Lohumi 2012).

Niosomes are non-ionic surfactant based, bi-layered multilamellar or unilamellar structures ranging in size from 10 to 10,000 nm (Ahmed 2013; Akbarzadeh et al. 2021). It forms only when surfactant and cholesterol are combined in the right ratio and the temperature is above the gel-liquid transition point (Bhardwaj et al. 2020). Niosomes can encapsulate both hydrophilic and hydrophobic drugs because of their special geometry. Niosomal formulation is essential for drug delivery and tumor targeting to create a stable, nontoxic, biodegradable, nonimmunogenic drug-loaded formula with good bioavailability (Masjedi and Montahaei 2021; Targhi et al. 2021). Niosomes also extend the time that the drug spends in the bloodstream (Faheela and Malathi 2022). They, therefore, exhibit promise as carriers for the sustained and/or controlled delivery of drugs to target sites (Amoabediny et al. 2018).

Radio-kinetic technique is used as a surrogate for liquid chromatography − mass spectrometry (LC–MS) to evaluate the pharmacokinetics of new drug dosage forms (Atzrodt et al. 2020; Jafarieh et al. 2015; Boseila et al. 2019; Rashed et al. 2017a; Ezz Eldin 2023). Major drawbacks of LC–MS include isobaric interferences, ion suppression effect, high cost and large samples number (Manohar and Marzinke 2016; Seger 2012). Radiokinetic studies allow following the radiolabeled drug biodistribution throughout the body and excretion over time. The radioactivity uptake, corresponding to drug uptake, in different body organs and fluids can be accurately tracked (Vogel et al. 2006, 1990; Ramakrishnan et al.; Rashed et al. 2017b). Iodine-131 is a radioisotope with a half-life of nearly 8 days and an energy of 364 keV of gamma radiation (Jimenez et al. 2019; Saha and Saha 2004). Such properties of ^131^I made it a suitable radionuclide to be applied in radiokinetic studies (Rashed et al. 2017b; Avcıbaşı et al. 2013; Bayrak et al. 2010).

In this research, niosomes were used as nanocarriers for MEF to increase its water solubility, stability and cancer targeting (Akbarzadeh et al. 2021). The Box-Behnken experimental design was used to investigate numerous niosomal formulation components to find the ideal formula. Fourier Transform Infrared Spectroscopy (FTIR), Transmission Electron Microscopy (TEM), Differential Scanning Calorimetry (DSC), ZP and EE % measures were performed for the formulated niosomes. In addition, ^131^I was used in the radiolabeling of MEF for the in vivo study to make a radiokinetic evaluation for MEF-loaded niosomes.

## Materials and methods

### Materials

Mefenamic acid and cholesterol were purchased from Sigma-Aldrich (St. Louis, MO, USA). A dialysis bag (12 kDa MWCO) bought from SERVAPOR^®^ Dialysis Membranes, Heidelberg, Germany. Tween 20, Span 20, disodium hydrogen phosphate, sodium dihydrogen phosphate, methanol and diethyl ether were purchased from ADWIC, El-Nasr Pharmaceutical Co., Egypt. All other reagents were of analytical grade. The Egyptian Atomic Energy Authority’s radioisotope production facility (RPF) provided iodine-131 (^131^I) as a no-carrier-added solution (Cairo, Egypt). Sigma Aldrich Chemical Co. (St. Louis, MO, USA) was the source of Chloramine-T (CAT).

### Methods

#### Preparation of MEF-loaded niosomes

Using the ether injection method, MEF-loaded niosomes were formulated. Methanol and diethyl ether (10 ml) with a ratio of 2.5:7.5 v/v were used as organic solvents to dissolve lipid (cholesterol), drug (MEF) and non-ionic surfactants (Span 20 and Tween 20 with a 1:1 (w/w) ratio). Then, organic solvent was injected slowly at a rate of 1 ml/min with a syringe into phosphate buffer solution (PBS) pH 7.4, as an aqueous solution that was preheated at 60 °C. According to the design, the buffer volume added was 10, 15, or 20 ml. This formulation was stirred for 30 min using a magnetic stirrer. Complete organic solvent evaporation was achieved at approximately 60 °C. The gradual addition of the lipid solution into the aqueous phase caused rapid ether vaporization due to the temperature differences between the two phases, which led to spontaneous vesiculation and the formation of niosomes. For additional investigation, the prepared niosomal formulations were kept refrigerated (Kulkarni et al. 2021; Yasamineh et al. 1878).

#### Box-Behnken experimental design

In this research, different formulations were created using a Box-Behnken design (BBD) for three factors, resulting in 15 experimental runs. Table 1 lists variables (dependent and independent), their respective levels, and the desired results. The cholesterol amount (A), surfactant amount (B), and hydration volume (C) were selected as independent variables (factors). To investigate their impact on dependent variables (responses) including: (Y1) vesicle size, (Y2) zeta potential, and (Y3) entrapment efficiency%. Design Expert^®^ (Version 12, Stat-Ease, Inc. USA) was utilized to examine three levels for each factor (low, medium, and high) and complete the statistical optimization process. Response surface methodology including ANOVA, multiple regression analysis, and statistical optimization were carried out using the software.

**Table 1:** Independent variables with their levels and dependent variables with desired values of the Box-Behnken design

Independent variables				
Factor coding	Name	Low level	Medium level	High level
A	Cholesterol amount (mg)	50.00	125.00	200.00
B	Surfactant amount (mg)	100.00	150.00	200.00
C	Hydration volume (ml)	10.00	15.00	20.00

Dependent variables			
Responses		Unit	Constraints
Y1	Particle size	nm	Minimum
Y2	Zeta potential	mv	Minimum
Y3	Entrapment efficiency	%	Maximum

#### Particle size

Dynamic light scattering (DLS) was used to evaluate the vesicle size of MEF-loaded niosomes at 25 °C using the Malvern Zetasizer (Nano ZS, Malvern, UK). The niosomal suspension was sonicated for 4 min using a water bath sonicatior then diluted (100 ×) with distilled water and vortexed for 3 min prior to every measuring. Each measurement repeated three times (Allam et al. 2021).

#### Zeta potential measurement

Zeta potential for niosomal preparations was measured in a zeta sizer (Malvern equipment, UK) using the Laser Doppler Micro-electrophoresis technique. Samples were put in single-use zeta cells after being diluted with distilled water. For each sample, measurements were performed three times, and average values ± SD were assessed (Shewaiter et al. 2021).

#### Entrapment efficiency

The quantity of the entrapped MEF in the MEF-loaded niosomes was evaluated with cooling centrifuge equipment (Remi C -24, Mumbai, India). Firstly, centrifugation of one ml of MEF-loaded niosomes for 1 h at 14,000 rpm was carried out. The supernatant containing unentrapped drug (0.1 ml) was then diluted with PBS pH 7.4 (5 ml) and tested in a UV–visible spectrophotometer against a blank (phosphate buffer). At a UV wavelength of 285 nm in a spectrophotometer (Shimadzu- UV, 1601 PC), sample absorbance was investigated for the determination of the quantity of encapsulated drug (Asaithambi et al. 2020). Entrapment efficiency (EE%) was assessed using the equation shown below (Haroun et al. 2022):

1 $$\mathrm{EE}\,(\mathrm{\%})=\frac{\mathrm{ Total \,quantity \,of \,drug }-\mathrm{ unentrapped \,drug}}{\mathrm{ Total \,quantity \,of \,drug}} \times 100$$

#### Optimization of MEF niosomes

To create a MEF niosomal formulation with the greatest desirable responses, formulation parameters were numerically and graphically optimized using Design Expert^®^ 12 software. The goal was to produce niosomes with the smallest vesicle size, the best and greatest zeta potential value (ZP), and the highest entrapment efficiency (EE%). The optimal formula, which had the greatest desirability, was chosen for further investigations.

#### Morphology of niosomes

To study the morphology of niosomes, TEM test was used. Before imaging, a droplet of the optimal formula was dropped on carbon grids that had been coated with copper, dyed by 2% (w/v) phosphotungstic acid, and then allowed for air drying. Using an 80 kV acceleration voltage, the formula was investigated through the TEM (JEM-100-CX11, Tokyo, Japan) (Mousa et al. 2022; Thabet et al. 2022).

#### Fourier transform infrared spectroscopy (FTIR)

To discover if there was any interaction between the excipients or polymers used to formulate niosomes and the drug, drug-excipient compatibility experiments were conducted. FTIR spectroscopy was used to determine if MEF and excipients being used were compatible (Manohar and Marzinke 2016). The dispersion method of potassium bromide (KBr) was used to determine the infrared spectra of MEF using a Fourier Transform Infrared Spectrophotometer (FTIR-4100 JASCO, Japan). Using dried KBr, the baseline correction was completed. After being oven-dried for 30 min, the sample to be tested and the KBr were completely blended at a ratio of 1:300 in a glass mortar. In a sample holder, these samples were scanned at a resolution of 2 cm^−1^ from 4000 to 400 cm^−1^ (Hassan et al. 2021; Chaudhari and Desai 2019).

#### Differential scanning calorimetry (DSC)

The DSC experiment was carried out using a differential scanning calorimeter (DSC-60, SHIMADZU). The MEF pure powder, physical drug mixture with excipients, plain niosomes and the optimal MEF niosomal formula were investigated. All samples were carefully weighed (about 2–3 mg) and then loaded in closed aluminum pans, where they were heated under 30 ml/min nitrogen gas flow from 40 to 200 °C at a rate of 10 °C/min. As a reference, an unfilled aluminum pan was utilized (Nishu et al. 2018).

#### Niosomes stability

The optimal MEF-loaded niosomes were split into two groups and their stability was investigated over a period of three months at 4 °C and 25 °C. The optimum formulation was placed in 20 ml glass vials and stored at the previously mentioned conditions. Zeta potential, entrapment efficiency and particle size were evaluated as stability indicating parameters at a specific time (0 and 90 days). Additionally, a visual examination of the two groups of the optimum formula was performed to evaluate the probability of color changes and sedimentation (Kulkarni et al. 2021; Khan et al. 2016).

#### In vitro drug release study

In comparison to the release of MEF from suspension, the MEF release from the optimal niosomal formulation was assessed. The dialysis method is the technique of choice for assessing the criteria of drug release from the niosomal formula. MEF release from niosomal formulation and suspension was monitored using a dialysis bag (MWCO12000 Da) at 37 °C for 24 h versus PBS (pH 7.4) to simulate the biological conditions. The optimal niosomes formula and suspension (1 ml of each) were loaded separately inside the dialysis tube, and the released quantity of MEF was evaluated in 100 ml of PBS with continuous stirring. At predefined intervals, (1, 2, 3, 4, 5, 6, 8, and 24 h), 2 ml of freshly formed PBS was added to replace two ml that were withdrawn from the release medium to assess the drug release rate. Lastly, the released concentrations of MEF were determined by examining the samples at 285 nm wavelength using a UV–visible spectrophotometer. This study was performed in triplicate (Targhi et al. 2021).

The mathematical methodology utilized to determine the kinetics of the total released percentages (cumulative) of MEF from the niosomes formulation was linear regression analysis. These models include Higuchi, Korsmeyer Peppas, First Order, and Zero Order. In the end, the correlation coefficient values were compared to select the model that best fit the data. The model with a correlation coefficient (R^2^) near to 1 would be chosen for drug release kinetics.

#### Radio-iodination of MEF using Iodine-131 (^131^I)

An oxidizing agent, Chloramine-T (CAT), was utilized in this study to radiolabel MEF with ^131^I through a direct electrophilic substitution reaction. An amber-colored vial containing the required quantity of MEF was filled with the proper quantity of CAT (dissolved in 70% ethanol). Under vigorous stirring, about 18–37 MBq of ^131^I were added to this reaction vial. The reaction of MEF labeling was run under various time intervals and different pH (5–9). By adding a droplet of sodium metabisulfite solution (10 mg/ml H_2_O), the reaction was stopped because of the extra amount of iodine (I_2_) was changed to iodide (I^−^) form. Ascending paper chromatography was used to quantify the radiochemical yield % of ^131^I-MEF (Ahmed et al. 2020; Aboumanei and Mahmoud 2022).

##### Paper Chromatographic Analysis of ^131^I-MEF

The ^131^I-MEF’s labeling efficiency was estimated by Paper Chromatography using 1 cm in width and 13 cm in length sheet of Whatman paper. Briefly, 1 to 2 µl of the reaction mixture was dropped at a distance of 1 cm from the lower edge of the sheet. Chloroform and methanol mixture with a ratio of 3:1 v/v was used as the mobile phase. When the mobile phase had totally developed, the sheet was dried and cut into small slices (1 cm length). Each piece was counted with a Scalar Ratemeter SR7 gamma counter (Nuclear Enterprises Ltd., USA). The ^131^I-MEF was assessed at* R*_f_ = 1, whereas at* R*_f_ = 0–0.1 free radioiodine settles (Ahmed et al. 2020; Aboumanei and Mahmoud 2022). To calculate the radiochemical yield percentage (RCY %), we applied the following equation:

2 $$\mathrm{Radiochemical \,yield }\,(\mathrm{\%})=\frac{\mathrm{Fraction }\,{of }^{131}\mathrm{I}-\mathrm{MEF}}{\mathrm{ Total \,activity }} \times 100$$

We investigated and assessed how various reaction conditions affected the efficiency of radioiodination to increase the radiochemical yield. These factors included the quantity of MEF, the reaction time, the reaction pH, and the oxidizing agent (CAT) amount.

##### In vitro stability of ^131^I-MEF

During the 24-h period, at various time intervals, samples (about 1–2 µl) were withdrawn from the prepared ^131^I-MEF solution and assessed for RCY % (Rashed et al. 2014).

##### Preparation of ^131^I-MEF loaded niosomes

The ^131^I-MEF niosomal formula was made using the prepared ^131^I-MEF complex in the same way as the optimum MEF niosomal formula (Ahmed et al. 2020; Aboumanei and Mahmoud 2022).

#### Biodistribution study

For in vivo biodistribution studies in mice, the radiokinetic technique was employed to monitor the effectiveness of the prepared MEF-loaded niosomes in targeting tumor sites in comparison to MEF solution. The animal ethics committee of the Egyptian Atomic Energy Authority approved this experiment. Swiss albino mice, with 20–25 g weight, were utilized in all experiments. Mice were divided into groups and provided with water and food throughout the experiment period.

##### Tumor induction in mice

In order to generate solid tumor in mice, Ehrlich ascites carcinoma cell line diluted with sterilized saline solution was used. Swiss albino male mice were injected carefully into their right thigh muscles with 0.2 ml of the previous solution. After 7–10 days, a detectable solid tumor developed in the right thigh muscle of the previously injected mice (Eldin et al. 2022; Nasr et al. 2018).

##### Biodistribution of ^131^I-MEF niosomes in tumor-bearing mice

Solid tumor-bearing mice were separated into two groups, A and B. Aliquots of 150 μl of ^131^I-MEF niosomes and ^131^I-MEF solution containing 5.2 MBq were injected intravenously into the two groups A and B, respectively. Mice injection was followed by dissection of anesthetized mice (*n* = 3) at different time interval post injection (0.5, 1, 2, 3, 4, 6 and 24 h). Bone, muscle and fresh blood samples were taken, weighted and counted for their radioactivity content. The percentages of blood, bone, and muscle mass in comparison with the total body weight were measured to be 7, 10 and 40%, respectively (Rashed et al. 2018; Sakr et al. 2017). The tumor muscle was totally separated from the mouse leg, rinsed with physiological saline, and then allowed to dry before being weighted. The contralateral muscle served as a normal control muscle to estimate the radiolabeled formula uptake into the tumor muscle (Haroun et al. 2022; Aboud et al. 2022). The other tissues and organs’ radioactivity were measured with a gamma counter. A group of three mice was used to determine the radioactivity uptake for each point of time, and the percent of injected dosage per gram (% ID/g ± S.D.) was reported for each organ (El-Sharawy et al. 2022; Khedr et al. 2019).

##### Statistical analysis

The maximal plasma concentration of ^131^I-MEF (C_max_) and the time to achieve it (T_max_) were simply calculated by graphing the average ^131^I-MEF uptake (% ID/g) in samples of blood and various tissues against time (hr). The area under the curve from 0 to 24 h (AUC_0–24_ h, % ID/g), the tumor AUC value (AUC_0–24_ h), the tumor drug targeting efficiency (AUC_target_ /AUC_non-target_) and the time to achieve half plasma concentration (T_1/2_, hr) were also reported.

All data of statistics were presented as means value ± SD. The statistical analysis was performed using the Design Expert^®^ program. Tukey’s post hoc assessment following a one-way ANOVA was applied, where the *p* value less than 0.05 was regarded as statistically significant.

## Results

### MEF-loaded niosomes optimization

**Fig. 1:**
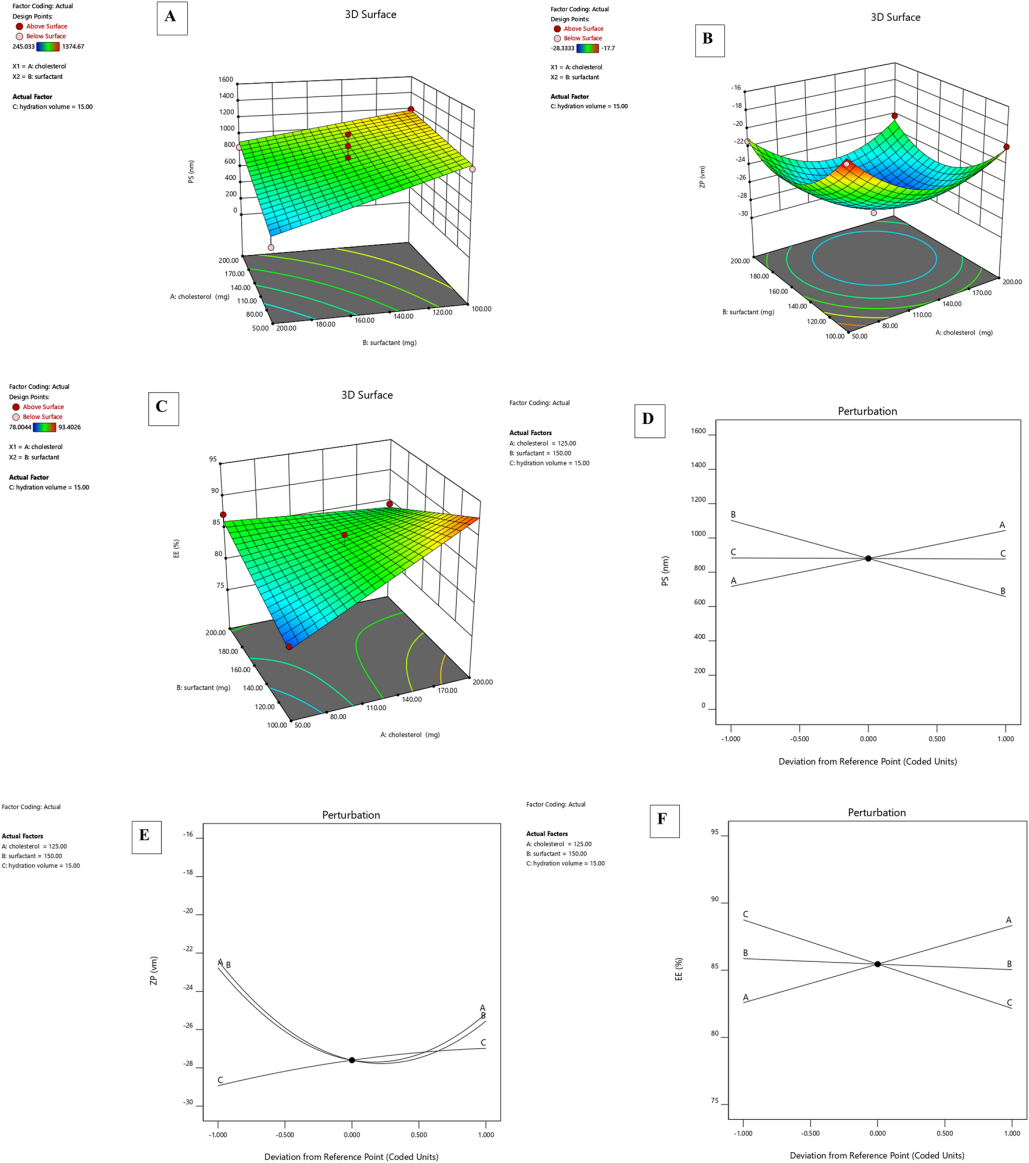
Response surface plots for **A** particle size, **B** zeta potential and **C** entrapment efficiency. Three-factor interaction plots **D** particle size, **E** zeta potential and **F** entrapment efficiency

The effect of independent factors (A- Cholesterol amount (mg), B- Surfactant amount (mg) and C- Hydration volume (ml)) on dependent responses (Y1-PS, Y2-ZP and Y3- EE %) was studied for optimization of MEF-loaded niosomes (Fig. 1). Box–Behnken design (Design-Expert^®^ Software version 12.0.1) was used for this optimization study.

Fifteen batches of MEF-loaded niosomes with three center points were prepared and tested according to the experimental design [Table 2]. The optimized MEF niosomal formula, which Design-Expert^®^ Software selected based on desirability value, was used for additional studies and characterizations.

**Table 2:** Composition of niosomal formulations and observed responses (means ± SD)

Run	A:cholesterol amount	B:surfactant amount	C:hydration time	PS	ZP	EE
	mg	mg	min	nm	mv	%
1	200	100	15	1195.4 ± 3.1	− 20.13 ± 1.1	71.39 ± 2.4
2	50	150	20	1142.3 ± 4.7	− 18.8 ± 0.9	78.00 ± 2.7
3	50	100	15	976.6 ± 1.4	− 17.7 ± 1.3	79.58 ± 3.1
4	125	100	10	1255.33 ± 5.2	− 23.4 ± 1.5	93.40 ± 1.6
5	125	100	20	849.7 ± 3.6	− 21.8 ± 0.7	90.41 ± 2.1
6	200	150	10	1374.66 ± 4.4	− 24.63 ± 1.3	90.83 ± 1.8
7	50	200	15	295.55 ± 1.2	− 21.33 ± 1.7	87.20 ± 3.7
8	125	200	20	180.8 ± 1.6	− 25.63 ± 1.4	85.38 ± 2.6
9	50	150	10	245.03 ± 1.8	− 26.7 ± 2.1	86.72 ± 3.9
10	125	200	10	531 ± 2.3	− 26.5 ± 1.8	83.66 ± 2.3
11	200	200	15	838.56 ± 3.7	− 22.2 ± 1.2	84.52 ± 1.9
12	125	150	15	1169.33 ± 6.4	− 28.33 ± 2.7	83.53 ± 3.3
13	125	150	15	904.16 ± 4.1	− 26.86 ± 2.9	84.25 ± 4.2
14	200	150	20	558.83 ± 2.9	− 27.16 ± 3.2	86.47 ± 1.5
15	125	150	15	1037 ± 5.2	− 27.6 ± 2.4	87.15 ± 0.6

### Particle size

Table 2 displays particle size measurement results of the prepared niosomes. The particle size of the samples ranged between 245.03 and 1374.66 nm. The PS of niosomes was affected by many factors, such as the cholesterol amount in the formula. The results indicated that the niosomal size increased with increasing the cholesterol amount (*p* = 0.0164). This could be due to the increased hydrophobicity of the bilayer caused by higher cholesterol levels leading to MEF sequestering in the hydrophobic bilayer (Witika and Walker 2021). This result is in agreement with previous findings by Moghddam et al. (Moghddam et al. 2016). The vesicles’ formation and properties are well known to be affected by the HLB of the used surfactant. Two classes of surfactants were used, Span 20 and Tween 20, to decrease the nonionic surfactants’ hydrophilic head group effect on the vesicles’ properties. Generally, preparing niosomes using polysorbate 20 (Tween 20) produces much larger particles than those prepared using sorbitan esters (Spans) due to the high HLB value of this polysorbate surfactant. In this study, the increase in surfactant amount resulted in a decreasing PS that may be due to using a combined mixture of surfactants (Tween 20 with Span 20) (*p* = 0.007) (Witika and Walker 2021).

The best-fit model for the niosomal particle size analysis was the 2FI model with a significant *p* value of 0.0026. The *P*-value of lack of fit was 1.33 (not significant), and the predicted and adjusted R-squared difference was < 0.2, indicating a good fit.

Final equation in terms of coded factors:

$${\text{PS}}\, = \,\, + \,{88}0.{31}\, + \,{163}.{5}0 \, *{\text{A }} - {222}.{6}0*{\text{ B }} - {3}.0{1}*{\text{C}}\, + \,{81}.0{5 }*{\text{A}}*{\text{B}} - {428}.{28}*{\text{A}}*{\text{C}}\, + \,{176}.{45}*{\text{B}}*{\text{C}}.$$

Polydispersity index (PDI) of niosomes ranged between 0.2 and 0.8, indicating that the produced niosomes were uniform in size and homogeneous. A PDI ≤ 0.5 is regarded as suitable for drug delivery applications because it demonstrates a relatively homogeneous and uniform distribution of nanocarriers. To provide proper particle distribution, the optimal formulation must have a PDI value ≤ 0.5.

### Zeta potential

Niosomes were physically stable since the ZP values of all formulations ranged from − 28.3 to − 17.7 mV (Allam et al. 2021; Ghanem et al. 2021). Cholesterol had a significant effect on ZP with a *p* value of 0.0094. Zeta potential increased, as an absolute value, by increasing the cholesterol amount leading to enhancing niosomes stability. As a result, cholesterol is an important excipient in the preparation of niosomes because it enhances the stability of niosomes’ bilayers and reduces drug leakage due to the retarded solute permeability of these vesicles’ aqueous core (Auda et al. 2016).

In addition, ZP increased by raising the surfactant amount until reaching a particular level, after this level, the ZP decreased. This could be because the hydrophilic–lipophilic balance (HLB) influenced ZP values. Because Span 20 (HLB 8.6) has a higher hydrophilic content than the other surfactants, it may have resulted in fewer counter ions adsorbed on the vesicles’ surface, leading to a rise in the ZP value at high surfactant amount (Abou-Taleb et al. 2018). Moreover, ZP decreased with increasing hydration volume, with a* p* value < 0.0206.

Final equation in terms of coded factors:

$${\text{ZP}}\, = \, - {27}.{6}0 \, - {1}.{2}0 \, *{\text{ A }} - {1}.{58 }*{\text{ B}}\, + \,0.{98 }*{\text{ C}}\, + \,0.{39 }*{\text{ A }}*{\text{ B }} - {2}.{61 }*{\text{ A }}*{\text{ C }} - 0.{18 }*{\text{ B }}*{\text{ C}}\, + \,{3}.{63 }*{\text{ A2}}\, + \,{3}.{63 }*{\text{ B2 }} - 0.{36 }*{\text{ C2}}.$$

### Entrapment efficiency

There was a decrease in EE% observed with an increase in the amount of surfactant. This may be due to the incorporation of a surfactant with a high HLB value, such as Tween 20 (HLB 16.7), into niosomes which decreased the hydrophobicity of the system and consequently decreased the EE% of MEF (Sharma et al. 2021). However, high entrapment efficiency ranged from 78 to 93.4% was due to the inclusion of Span 20 in formulations (Witika and Walker 2021). Cholesterol addition led to an increase in the amount of MEF being sequestered in the hydrophobic bilayer and a subsequent increase in EE% (Witika and Walker 2021; Sharma et al. 2021).

The ideal volume of hydration media was ten ml which effectively hydrated the produced film. In this study, larger volumes were found to have a negative effect on EE%. That was explained by the fact that increasing the drug dispersion by increasing the hydration medium volume results in small vesicles with a low EE% (Aziz et al. 2018).

Final equation in terms of coded factors:

$${\text{EE}}\, = \,\, + \,{85}.{45}\, + \,{2}.{87 }*{\text{ A }} - 0.{41}*{\text{ B }} - {3}.{3}0*{\text{ C }} - {3}.{94}*{\text{A}}*{\text{B}}\, + \,{1}.0{9 }*{\text{A}}*{\text{C}}\, + \,{4}.{18}*{\text{ B}}*{\text{C}}.$$

### Selection of optimal formulation

All models were statistically significant, as indicated in Table 3. The Y1 and Y3 factors had a 2FI model fit, whereas the Y2 factor had a Quadratic model fit, as presented in Table 3.

**Table 3:** Responses of regression analysis for particle size (PS), Zeta Potential (ZP) and %Entrapment Efficency (%EE)

Response	*R*^2^	Adjusted *R*^2^	Predicted *R*^2^	Adequate precision	Minimum	Maximum	*p* value	Lack of fit	Trans	Model
Y1 = PS (nm)	0.9075	0.8283	0.7314	11.357	180.80	1374.666	0.0026	0.4818	None	2FI
Y2 = ZP (mv)	0.9790	0.9411	0.7540	15.395	− 28.333	− 17.7	0.0011	0.4299	None	Quadratic
Y3 = EE (%)	0.9468	0.8937	0.8180	15.524	78.0044	93.402	0.0014	0.9040	None	2FI

According to the design optimization, the optimal formula with the highest desirability value (0.819) was prepared with the following composition (170.207 mg surfactant, 67.716 mg cholesterol, and a 10 ml hydration volume). This selected optimum formula had a vesicle size of 247.23 nm, a zeta potential of -28.3 mV, a PDI of 0.44, and an entrapment efficiency of 85.81% (Table 4).

**Table 4:** Composition, actual and predicted responses for the optimum MEF niosomes

Factor optimized level	
X1 = cholesterol amount	67.716 mg
X2 = surfactant amount	170.207 mg
X3 = hydration volume	10 ml

Response	Predicted	Observed	Residual
Y1 = PS (nm)	245.033	247.23 ± 1.3	− 2.197
Y2 = ZP (mV)	− 27.987	− 28.3 ± 2.1	0.313
Y3 = EE (%)	86.752	85.81 ± 1.7	0.942

### Transmission electron microscopy (TEM)

**Fig. 2:**
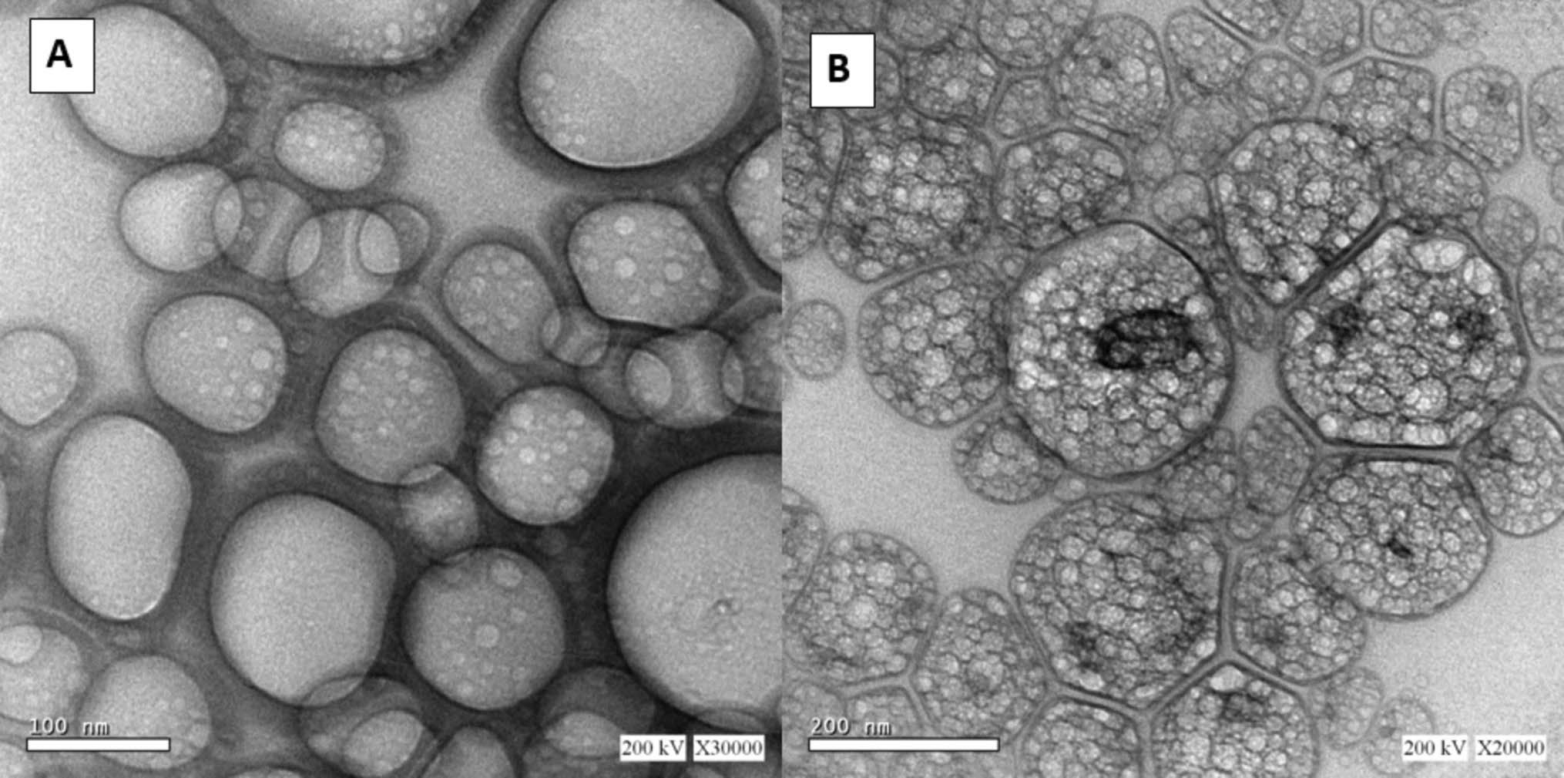
Transmission electron microscopy of optimum niosomal formulation: **A** 30,000 × and **B** 20,000 × magnification

TEM is frequently used to examine the morphology of the niosomal formulation. The MEF niosomal formulation panoramic image showed niosomes vesicles with a spherical shape without aggregation at 30,000× (Fig. 2A) and 20,000× (Fig. 2B) magnification. The image obtained by TEM of MEF niosomes presented spherical vesicles with a size of around 100–200 nm that was similar to the size obtained via the DLS experiment (de Siqueira et al. , 2022). Additionally, TEM scans demonstrated that MEF-loaded niosomes appeared as distributed spherical particles and had a hollow vesicular shape with well-defined edges (Shilakari Asthana et al. 2016).

### FTIR study

**Fig. 3:**
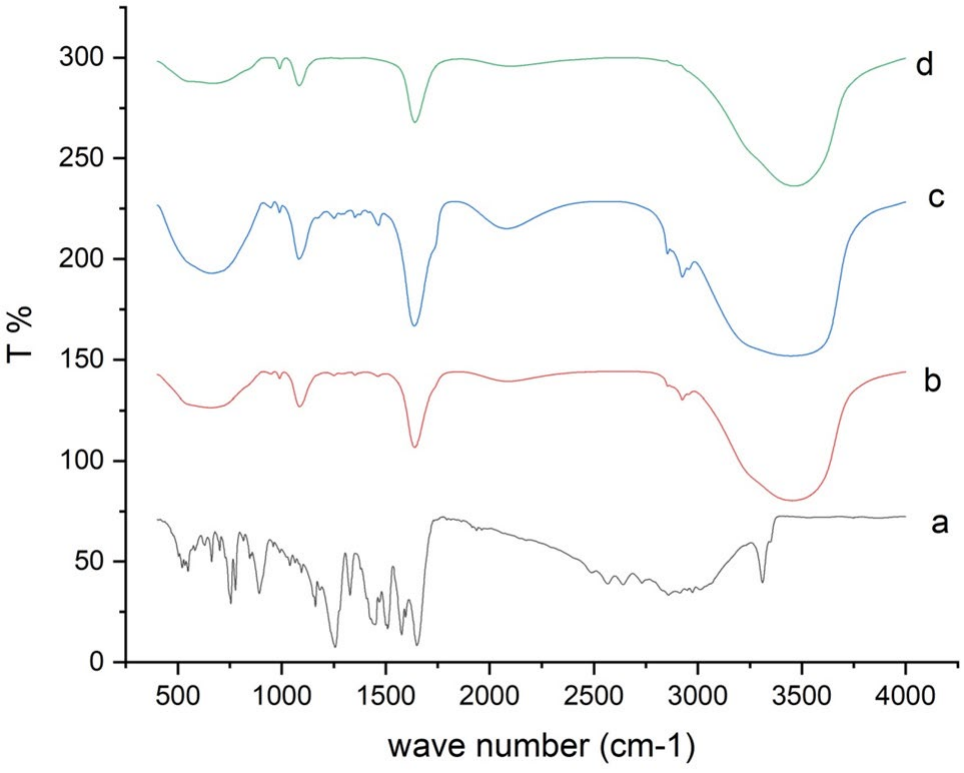
Fourier Transform Infrared Spectra of **a** MEF, **b** plain niosomes, **c** physical mixture and **d** MEF-loaded niosomes

MEF had a secondary amine characteristic peak at 3310 cm^−1^ in its FTIR spectrum (Fig. 3). Due to the presence of the –OH group, there was a broad band in the range of 3010–2858 cm^−1^. The –OH group also represented intra- and intermolecular hydrogen bonding, which overlapped with the (–CH_3_) group. The presence of the peak at 1649–1733 cm^−1^ was due to the appearance of the C=O group. The phenyl group presence is indicated by a peak presence at 1076 cm^−1^. In the formula, the secondary amine group was presented at 3455 cm^−1^ and also overlapped with the (–OH) group. The C=O of the carboxyl salt was presented at 1641 cm^−1^ and the phenyl group was presented at 1084 cm^−1^ (Bhardwaj et al. 2015; Puspaningtyas 2017).

As a result, all the characteristic peaks of the drug were presented and there were no noticeable additional peaks or peak shifts of MEF detected in the formula. Such results indicated that MEF had not been exposed to chemical degradation or lost compatibility in the presence of the additives. Therefore, FTIR confirmed the stability of the niosomal formula.

### Differential scanning calorimetry (DSC)

**Fig. 4:**
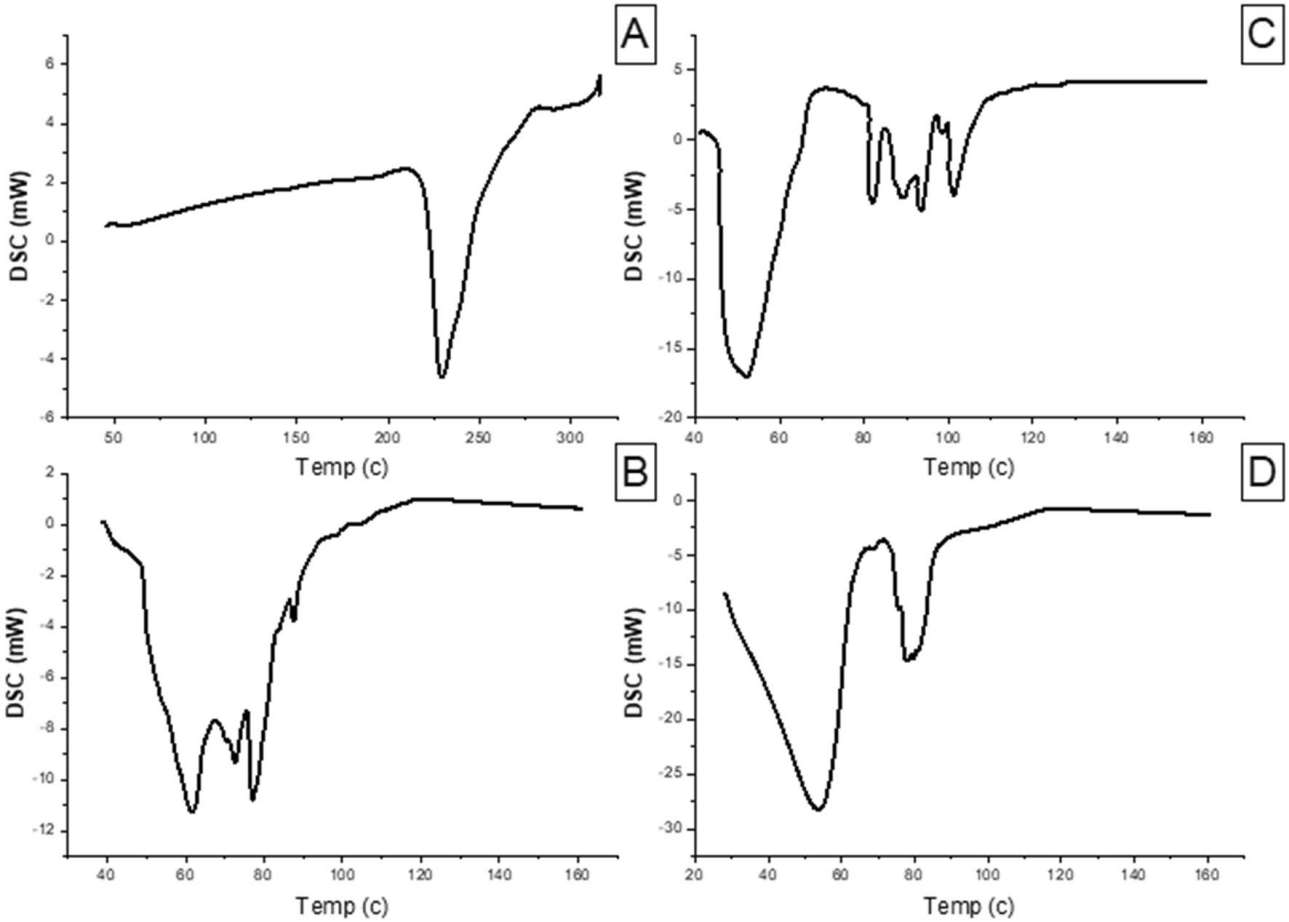
Differential scanning calorimetry of **A** MEF, **B** plain niosomes, **C** physical mixture and **D** MEF-loaded niosomes

DSC was applied at a temperature range of 40–300 °C to investigate if the niosomal formulation has any excipients that are incompatible with MEF. The obtained thermogram presented an endothermic peak for MEF at 229.3 °C. The crystalline peak of MEF disappeared in the niosomal formula curve. The absence of a distinctive MEF peak indicates that the drug was fully dissolved in the niosomal system and converted to an amorphous form. In addition to that the thermogram curves showed that the additives and the drug were compatible with each other (Utami et al. 2017) (Fig. 4).

### Stability study

After 3 months of storage, the niosomal formulations showed no change in color. They kept their homogeneous, opaque white appearance and there wasn’t any precipitation. To obtain more detailed knowledge about the niosomes’ stability, any changes in zeta potential, vesicle size and EE% were used as stability-indicating factors (Temprom et al. 2022; Ghafelehbashi et al. 2019). These variables were assessed at predetermined intervals. According to Table 5, it could be seen that the MEF niosomal formula showed a small change in vesicle size during the 3 months of storage. Under refrigeration at 4 °C, the distribution of vesicle size changed by an insignificant value due to the existence of the appropriate electrostatic repulsion force, which prevents agglomeration. However, at room temperature (25 ± 2 °C), the niosomal formula was found to aggregate, indicating that the fluidity of the bilayer membrane increased at higher temperatures (Kulkarni et al. 2021). Because the lipophilic part rigidity in the niosomes rises at lower temperatures, the sample held at 4 °C was more stable than that kept at 25 °C (Sayyad et al. 2021). Another essential stability parameter is the niosomes’ capability to retain the drug in its encapsulated form during the storage period. The entrapment efficiency percentage decreased from 85.81% to 81.62%, after three months of storage at 25 °C. This was due to the surfactant reaching its superior alternation temperature, which resulted in the formation of a more stable membrane. Such stability data suggested that these formulations were more stable at low temperatures (Ghafelehbashi et al. 2019).

**Table 5:** Stability of the optimized MEF-loaded niosomes

	PS (nm)	ZP (mV)	EE%
At zero day	247.23 ± 1.3	– 28.3 ± 2.1	85.81 ± 1.7
After 90 days at 4 °C	250.9 ± 1.7	– 27.5 ± 1.9	84.98 ± 2.6
After 90 days at 25 °C	274.2 ± 2.4	– 24.2 ± 1.5	81.62 ± 2.2

### In vitro release study

**Fig. 5:**
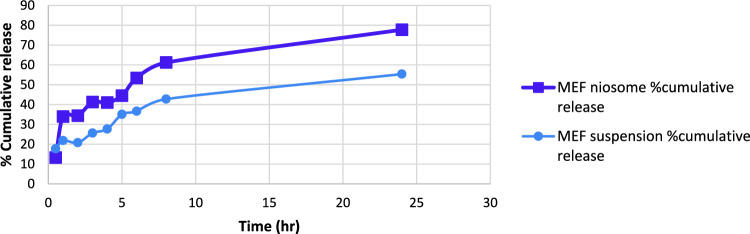
In vitro release of MEF suspension and optimal MEF niosomes (cumulative release % ± SD)

A quick initial drug release was followed by a prolonged release phase (Fig. 5) indicating a biphasic cumulative release pattern for MEF. The quick burst release could be due to the drug desorption from the niosomes surface. Drug diffusion across vesicular bilayers may be responsible for the slower phase, indicating that the entrapped drug was relatively stable. Niosomes were good drug carrier with good release since MEF was released cumulatively from suspension and niosomes formulation at rates of 55.36% and 77.73%, respectively, after 24 h (Targhi et al. 2021).

The in vitro release data gained from the optimal niosomal formulation was fitted to different kinetic models to predict the mechanism of MEF release from the niosomes. Table 6 displays the regression coefficient (*R*^2^) values for each model. The results showed that Higuchi’s model best explained niosomal formulation release, indicating that the drug diffused more slowly as the distance for diffusion increased. So, the diffusion mechanism controlled the release process of MEF from the niosomal formula suggesting a matrix diffusion-based release, which was unpredicted with such a liquid formula (Temprom et al. 2022). The responsibility for this behavior lies in the fact that the lipophilic drug was trapped within the lipid bilayer of the produced niosomes, despite the test system’s fluidity. Firstly, the drug was released quickly from the multi-lamellar structure’s surface layer of niosomes, followed by a diffusion-controlled release from the interior layers (Fayed et al. 2021).

**Table 6:** Different release kinetics* R*^2^ values for MEF suspension and optimal MEF niosomes

Formula	Zero (*R*^2^)	First (*R*^2^)	Diffusion (*R*^2^)	Krosmear (*R*^2^)	Hixon (*R*^2^)	Order of permeation
MEF niosomes	0.7552	0.4917	0.9016	0.8712	0.5856	Diffusion
MEF suspension	0.8398	0.7199	0.9442	0.9211	0.7622	Diffusion

### Radiolabeling of MEF using ^131^I

**Fig. 6:**
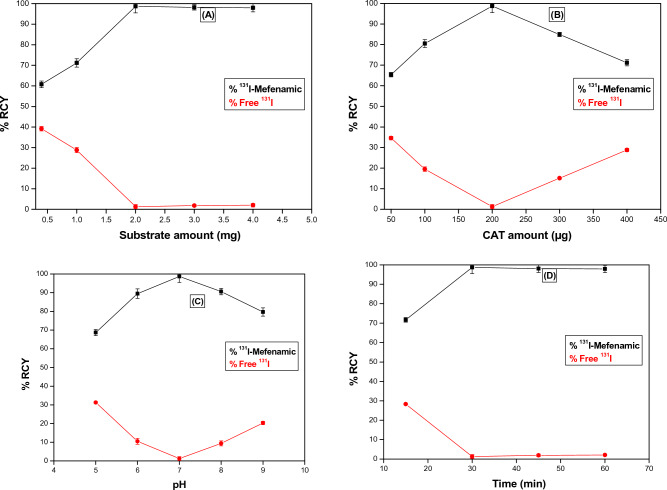
Influence of various radiolabeling parameters; **A** MEF quantity, **B** CAT quantity, **C** pH and **D** reaction time, on ^131^I-MEF radiochemical yield

Figure 6 presents how different factors of radiolabeling influenced the ^131^I-MEF niosomes RCY%. The highest RCY% of the ^131^I-MEF complex was 98.7 ± 0.8% was achieved using 200 µg of CAT, 18–37 MBq of ^131^I and 2 mg of MEF at 30 min reaction time, pH of 7 and room temperature (25 °C). In vitro, the ^131^I-MEF complex maintained its stability for at least 24 h. The physical and chemical characteristics of the niosomes, such as EE%, PS, PDI, ZP or in vitro release, were unaffected by the use of radiolabeled MEF in the niosomes’ creation (Ahmed et al. 2020).

### Biodistribution of ^131^I-MEF niosomes

The two groups of tumor-bearing mice (each consisting of 21 mice) were used to study the in vivo biodistribution of ^131^I-MEF niosomes. Groups A and B were intravenously (I.V) injected with ^131^I-MEF niosomes and ^131^I-MEF solution, respectively. Using three mice for each time interval, the radioactivity (measured as %ID/g) was calculated in different organs and body fluids during the 24-h period (Haroun et al. 2022).

**Fig. 7:**
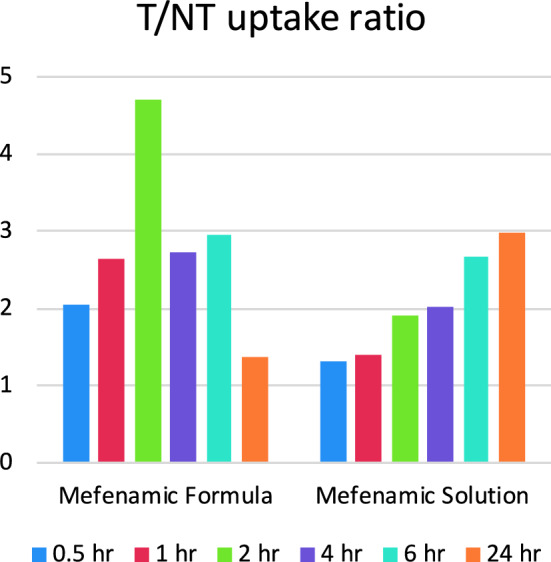
Target/non-target ratio (T/NT) for ^131^I-MEF niosomes and ^131^I-MEF solution at different time intervals post-injection

The accumulation of drug in tumor tissues started to rise within 60 min after I.V. injection, while the uptake of drug in the blood started to gradually decline. The enhanced permeability and retention (EPR) effect was the reason for enhancing the ^131^I-MEF niosomes’ accumulation and permeability into malignant cells. In addition, the nanoparticles (niosomes) were targeted to cancer tissues passively due to the small niosomal particle size. Particles with a small diameter avoided internalization into the Mononuclear Phagocyte System (MPS), resulting in a longer circulation time due to reduced clearance by macrophages. In comparison to group A, group B’s biodistribution profile showed less ^131^I-MEF accumulation at the tumor site. There were noticeable differences between groups A and B in tumor uptake of ^131^I-MEF and accumulation at the tumor site, as reported in Table 7. The highest accumulation of ^131^I-MEF at cancer cells in groups A and B post-I.V injection was 2.73% ID/g at 60 min and 1.94% ID/g at 30 min, respectively. Examination of the target (solid tumor) to non-target (normal muscle) ratio (T/NT) of ^131^I-MEF was an essential parameter (Fig. 7). Regarding group A, the highest T/NT ratio was attained after 2 h of the I.V injection, which was 4.692. However, it was only 2.978 in group B after 24 h post I.V. injection. The Tumor/Blood (T/B) ratio, which should be higher than 1, determines the potentiation of any novel nano-radiopharmaceutical (Witika and Walker 2021). The ^131^I-MEF niosomes had a T/B ratio greater than 1 at 1 and 2 h after I.V. injection (Table 7) (El-Safoury et al. 2021). The pharmacokinetic differences between ^131^I-MEF solution and ^131^I-MEF niosomes were mathematically calculated using C_max_, T_max_, T_1/2_, AUC_0–24_, tumor AUC_0–24_ and the tumor drug targeting efficiency (AUC_target_/AUC_non-target_) as shown in Table 8. Consequently, ^131^I-MEF niosomes improved MEF uptake in tumor tissues following intravenous injection and it could be considered as a hopeful nano-radiopharmaceutical that potentiates tumor treatment.

**Table 7:** Radioactivity uptake (%ID/g ± S.D) in tumor and normal muscle at different time intervals post-injection (hr), target/non-target ratio (T/NT) and target/blood ratio (T/B)

Organs and body fluids	Detected dose/gram % at different time intervals post-injection (hr)					
	0.5 h	1 h	2 h	4 h	6 h	24 h
^131^I-MEF niosomes						
Tumor (T)	2.176 ± 0.25	2.738 ± 0.16	1.607 ± 0.09	1.21 ± 0.14	1.053 ± 0.06	0.791 ± 0.07
Muscle (NT)	1.066 ± 0.19	1.039 ± 0.42	0.343 ± 0.26	0.446 ± 0.11	0.358 ± 0.32	0.581 ± 0.08
T/NT	2.041	2.635	4.692	2.713	2.941	1.363
T/B	0.486	1.107	1.152	0.840	0.941	0.505
^131^I-MEF solution						
Tumor (T)	1.94 ± 0.14	1.62 ± 0.27	1.47 ± 0.34	1.43 ± 0.19	1.39 ± 0.15	1.34 ± 0.18
Muscle (NT)	1.47 ± 0.12	1.17 ± 0.28	0.77 ± 0.16	0.71 ± 0.08	0.52 ± 0.01	0.45 ± 0.02
T/NT	1.32	1.385	1.909	2.014	2.673	2.978
T/B	0.144	0.360	0.369	0.475	0.556	0.720

**Table 8:** C_max_, T_max_, T_1/2,_ AUC_0–24,_ Tumor AUC_0–24_ and Tumor drug targeting efficiency for ^131^I-MEF niosomes and ^131^I-MEF solution

Formula	C_max_ (ID/g)	T_max_ (hr)	T_1/2_ (hr)	AUC_0–24_	Tumor AUC_0–24_	Tumor drug targeting efficiency (AUC_target_ /AUC_non-target_)
^131^I-MEF niosomes	4.471	0.5	0.213	59.079	42.3348	2.4691
^131^I-MEF solution	13.40	0.5	0.261	155.144	34.2033	1.4782

## Conclusion

By using the ether injection method, a niosomal formulation of MEF made of cholesterol and non-ionic surfactants (Tween 20 and Span 20) was created. DSC demonstrated that MEF was trapped in an amorphous form and that the nanovesicle size was less than 300 nm. After formulation, the drug’s molecular chemistry did not change, according to FTIR. The optimum niosomal formula of MEF displayed a high entrapment efficiency of 85.81%. The niosomal formula continuously released the drug in a sustained manner for 24 h. MEF was radiolabeled successfully by ^131^I with a radiochemical yield of 98.7%. The radiokinetic analysis revealed that passive targeting of the nanosized niosomes increased the delivery of MEF to the tumor cells. More importantly, optimum ^131^I-MEF niosomes showed promising properties and potentiated cancer activity due to enhanced targeting, encapsulation efficiency and release rate and showed a high accumulation in tumor tissues. In conclusion, the optimal niosomal formula of MEF was effective in improving MEF delivery as an anticancer agent. Further histopathology, pharmacodynamics and clinical studies are required to improve the findings of this research.
